# Decreased Systemic and Airway Sirtuin 1 Expression in Adults With Bronchiectasis

**DOI:** 10.3389/fmed.2021.768770

**Published:** 2022-01-06

**Authors:** Xiao-rong Han, Lai-jian Cen, Cui-xia Pan, Zhen-hong Lin, Hui-min Li, Ri-lan Zhang, Yan Huang, Yong-hua Gao, Wei-jie Guan

**Affiliations:** ^1^State Key Laboratory of Respiratory Disease, National Clinical Research Center for Respiratory Disease, Guangzhou Institute of Respiratory Health, The First Affiliated Hospital of Guangzhou Medical University, Guangzhou, China; ^2^Department of Respiratory and Critical Care Medicine, Shanghai Pulmonary Hospital, Tongji University School of Medicine, Shanghai, China; ^3^Department of Thoracic Surgery, Guangzhou Institute of Respiratory Disease, First Affiliated Hospital of Guangzhou Medical University, Guangzhou, China

**Keywords:** aging, bronchiectasis, sirtuin 1, senescence, disease severity, bronchial epithelium

## Abstract

**Aim:** Whether accelerated aging, reflected by sirtuin 1 (SIRT1) expression, is implicated in bronchiectasis remains largely unknown. We sought to determine the patterns of SIRT1 and other aging markers in systemic circulation and airways and their expression levels associated with bronchiectasis severity and exacerbation.

**Methods:** We enrolled 132 patients with bronchiectasis and 50 healthy subjects in a prospective cohort study to profile aging markers in systemic circulation and recruited 36 patients with bronchiectasis and 32 disease controls (idiopathic pulmonary fibrosis or tumors) in a cross-sectional study to profile aging markers in bronchial epithelium of both large-to-medium and small airways. We profiled aging marker expression from peripheral blood mononuclear cells and enumerated the positively stained cells for detection of aging marker expression in bronchial epithelium.

**Results:** Compared with healthy controls, the relative telomere length (median: 0.88 vs. 0.99, *p* = 0.009), SIRT1 (median: 0.89 vs. 0.99, *p* = 0.002), and Ku80 (median: 0.87 vs. 0.96, *p* < 0.001) expression levels were consistently lower in the peripheral blood mononuclear cells among patients with bronchiectasis and modestly discriminated patients with bronchiectasis from healthy controls. No remarkable changes in SIRT1, telomere length, or Ku70 were identified at onset of exacerbation. Within the bronchial epithelium, the percentage of positively stained cells was lower for SIRT1 (median: 25.1 vs. 57.2%, *p* < 0.05) and numerically lower for p16 (median: 40.0 vs. 45.1%) and p21 (median: 28.9 vs. 35.9%) in patients with bronchiectasis than in disease controls (*p* > 0.05).

**Conclusion:** SIRT1 was downregulated in systemic circulation and bronchiectatic airways, which was independent of disease severity and lung function impairment.

## Introduction

Bronchiectasis is a common chronic airway inflammatory disease characterized by irreversible bronchial dilatation (1). The prevalence was 67–566.1 per 100,000 inhabitants in Europe and North America and 1,200 per 100,000 inhabitants among people aged 40 years or greater in China (2, 3). The vicious cycle hypothesis (airway infection, inflammation, and destruction) remains central to guide clinical management (4), which typically consists of airway clearance and antibiotics (5).

Cells normally regenerate to repair the airway microenvironment in response to infections and inflammation. However, the defective repair associated with accelerated aging (i.e., telomere attrition, epigenetic modifications) reportedly predisposed to chronic obstructive pulmonary disease (COPD) and idiopathic pulmonary fibrosis (IPF) (6, 7). Accelerated aging, evidenced by telomere shortening, increased p21, and decreased sirtuin 1 (SIRT1) expression, has also been identified in large airways of patients with bronchiectasis (8).

Various markers reflect different aspects of aging. These generally include nicotinamide adenine dinucleotide-dependent deacetylase (i.e., SIRT1) and klotho, which confer antiaging effects and cyclin-dependent kinase inhibitor 1A (p21) and 2A (p16), which are cell cycle inhibitors (9–11). SIRT1 is a crucial member of the nicotinamide adenine dinucleotide-dependent deacetylase family via antiaging effects (12, 13). In murine models of COPD, upregulation of SIRT1 alleviated inflammation and altered the expression of markers reflecting various pathways of aging, including decreased SIRT1 expression, compared with healthy controls (14). These signals could be readily detected in peripheral blood mononuclear cells (PBMCs) (6, 7), which are a relatively non-invasive sample source that has rendered the evaluation of aging marker expression in patients with chronic airway diseases technically feasible in clinical practice.

The heterogeneity of bronchiectasis indicates that the prevailing vicious cycle hypothesis might not have fully accounted for the mechanisms underlying the pathogenesis. Given the abovementioned pilot findings, assessment of aging marker expression in adults with bronchiectasis might help to provide additional insights into the pathogenesis and therapeutic interventions.

Currently, aging marker expression has only been profiled in one single small study without delineating the association with the clinical meaningful metrics (8). In this regard, an integrated analysis of the aging marker expression in different compartments (e.g., systemic circulation, different anatomical locations of the lower airways) would be clinically more informative. We hypothesized that accelerated aging was implicated in bronchiectasis and correlated with bronchiectasis severity. In this study, we profiled SIRT1 and a panel of other aging markers in PBMCs and the bronchiectatic epithelium in adults with bronchiectasis.

## Methods

### Study Participants

We recruited participants aged 18–75 years for two substudies between June 2017 and January 2021. To profile marker expression in PBMCs (Study 1), we included symptomatic (i.e., cough, sputum production) patients with bronchiectasis whose diagnosis was based on chest high-resolution CT in the past 12 months. We excluded patients with bronchiectasis who had exacerbation (the 2017 *European Respiratory Society* expert consensus criteria) (15) or antibiotic use within 4 weeks before enrollment. We recruited healthy controls via advertisement who had normal chest X-ray and spirometry and were free from lower airway symptoms or severe systemic diseases. We excluded participants who were pregnant or breastfeeding and those who had limited understandings.

To characterize aging marker expression in bronchial epithelium (Study 2), we enrolled another cohort of patients with bronchiectasis (bronchiectasis group) and those with IPF or lung malignancy as disease controls. All the study participants in Study 2 were scheduled for segmentectomy or lobectomy.

The Ethics Committee of The First Affiliated Hospital of Guangzhou Medical University (Medical Ethics 2016, the 32th) approved for the study protocol. All the participants gave a written informed consent.

### Study Design

In Study 1, we collected the clinical history, exacerbation frequencies, smoking status, and concomitant medications from medical charts at the initial visits. Other data included the assessment of bronchiectasis etiology, radiological severity (modified Reiff score), spirometry, sputum bacteriology, and disease severity [the Bronchiectasis Severity Index (BSI) and the E-FACED score] (16–18). Meanwhile, patients donated 8 ml peripheral blood for marker profiling. To determine the variations in marker expression, we collected peripheral blood in a subgroup when clinically stable (scheduled at least 1 month apart) and in patients at onset of exacerbation (before antibiotics administration). Healthy controls attended the first single visit that consisted of clinical history and smoking status inquiry, chest X-ray, spirometry, and peripheral blood withdrawal only.

In Study 2 (cross-sectional), we sampled the paired bronchial epithelium from large-to-medium (the 3rd to 6th generation) and small airways (the 7th generation and beyond), via forceps excision, from hospitalized patients with bronchiectasis and disease controls. We did not sample large airway epithelium among patients who underwent segmentectomy. For disease controls who had malignancy, we sampled the bronchial epithelium at least 1 cm away from the border of tumor to minimize the impact on marker expression.

### Peripheral Blood Processing and Senescence Marker Profiling

We collected the fresh peripheral blood into an anticoagulant tube and processed within 12 h. We diluted the whole blood with an aliquot of phosphate buffer solution and mixed with the Human Leukocyte Separation Medium (Dakewe Incorporation, Shenzhen, China) for isolating the buffy coat (containing PBMCs), which was meticulously aspirated for storage in −80° freezers before analysis. We extracted the DNA for assessing the relative leukocyte telomere length (T/S ratio) and extracted the RNA and reverse transcribed for profiling other markers: (a) markers modulating telomere length [telomerase reverse transcriptase (TERT)]; (b) antiaging markers [SIRT1, soluble klotho (s-klotho), and total klotho (t-klotho)]; (c) cell senescence markers (p16 and p21); and (d) DNA repair markers (Ku70, Ku80, and TRF2). We profiled superoxide dismutase 2 (SOD2) and catalase expression because aging has been associated with oxidative stress responses. We used PCR to determine the expression levels of different markers and normalized with those of hypoxanthine phosphoribosyltransferase 1. Except for the relative telomere length, we calculated the ratio of the cycle threshold in patients with bronchiectasis (when clinically stable and at exacerbation) and in healthy controls to represent the relative expression for each marker. See *Online* Supplementary Material for details.

### Tissue Preparation and Immunohistochemistry Staining

We further verified SIRT1 (antiaging), p16, and p21 (cell senescence markers) expression in bronchial epithelium in light that they closely reflected the aging pathways and that staining could be achieved via immunohistochemistry. We incubated the pretreated sections with primary polyclonal antibodies of p21 (1:100, anti-p21, Abcam, Waltham, Massachusetts, USA), p16 (1:100, anti-p16, Abcam, Waltham, Massachusetts, USA), and SIRT1 (1:200, anti-SIRT1, Abcam, Waltham, Massachusetts, USA). We stained the slides with diaminobenzidine (1:50, Dako A/S, Denmark) at 37°C and washed thrice in Tris buffer solution. We processed negative controls without the primary antibody. We then counterstained the slides with hematoxylin. See *Online* Supplementary Material for details.

### Immunohistochemistry Outcome Assessment

We obtained images at 400X magnifications with the light microscopy (Olympus CKX53, Osaka, Japan). We randomly evaluated five high-power fields (by two independent reviewers blinded to the profiles of patient) through calculating the percentage of positively stained cells (with the cell nuclei expressing the marker) (8). We then averaged the mean reading of five fields. We adjudicated by means of consensus for any major disagreement (>10% difference) in the cell counts between two reviewers.

### Statistical Analysis

Although we cannot directly estimate the sample size due to the lack of literature report among patients with bronchiectasis, this sample size was comparable to a study characterizing patients with COPD (14). We performed analyses using the SPSS software version 22.0 (SPSS Incorporation, Chicago, Illinois, USA), the GraphPad Prism (GraphPad Incorporation, San Diego, California, USA), and the R software package. We verified normality for continuous variables and expressed as mean ± SD or median (interquartile range). We summarized categorical variables as count (percentage). We compared continuous variables with the Student's *t*-test or the non-parametric Mann–Whitney *U*-test and categorical variables with the chi-squared test or the Fisher's exact test as appropriate. To minimize confounding effects of the age, we adjusted with the lung age (19). We adopted the receiver operating characteristic curve to analyze the diagnostic performance of different markers (including their combination), which calculated the area under the curve (AUC) and 95% CI. We used the paired *t*-test to compare marker expression when clinically stable and at exacerbation. We applied the Bland–Altman plot to evaluate the concordance for two clinically stable visits. We conducted correlation analysis using the Pearson's or Spearman's model. We determined the association between marker expression and clinical variables with the univariate linear regression model, followed by the adjustment of age, the percentage predicted of forced expiratory volume in one second (FEV_1_ pred%), and the ratio of FEV_1_ and forced vital capacity (FEV_1_/FVC). Variables with *p*-value of 0.20 or less were entered into the multivariate regression model. To estimate whether baseline levels predicted risks of exacerbation, we calculated the hazards ratio by stratifying marker expression into the median.

## Results

### Study 1

#### Study Participants and Baseline Characteristics

**Figure 1:**
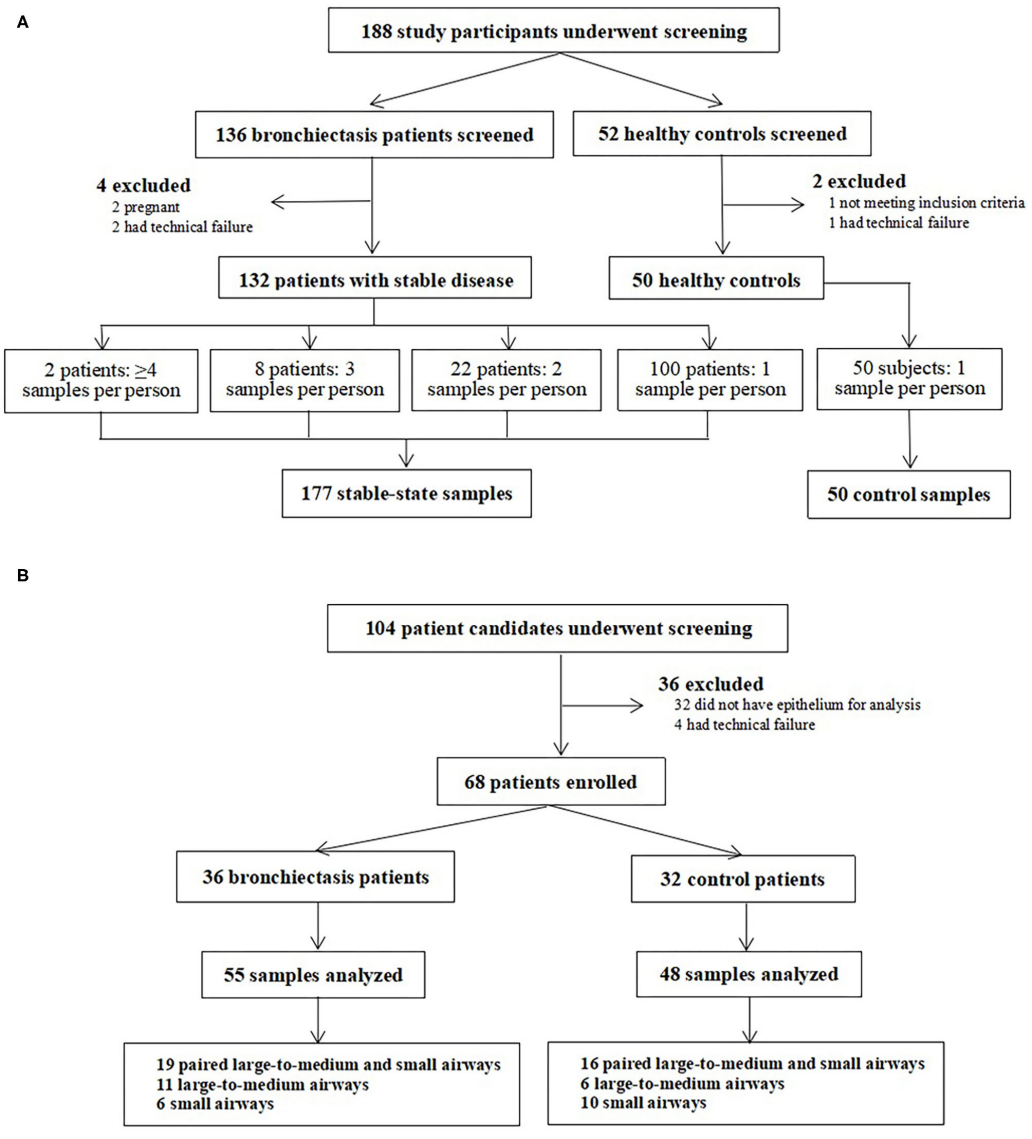
Recruitment flowchart of the study participants. **(A)** presents recruitment flowchart of the study participants for Study 1, where patients with clinically stable and exacerbation (in a subgroup of patients) of bronchiectasis as well as the healthy controls were included. **(B)** displays recruitment flowchart of the study participants for Study 2, where patients with bronchiectasis and the disease controls who were scheduled for elective lobectomy or segmentectomy were included.

Of the 188 participants who underwent screening between April 2019 and January 2021, two participants were pregnant, three participants were subjected to technical error in isolating PBMCs, and one participant failed quality check for spirometry. Finally, we included 132 patients with bronchiectasis and 50 healthy controls for analyses (Figure 1A). These patients donated 177 clinically stable PBMCs samples (mean: 1.3 per patient). A total of 22 patients donated dual samples, 8 patients donated triple samples, and one patient donated each quadruple and quintuple samples. In healthy controls, we collected a single PBMCs sample only. In total, 32 patients with bronchiectasis who attended exacerbation visits donated 36 samples.

The baseline characteristics are shown in Table 1. The median age was 49.0 years in patients with bronchiectasis and half were females. The most common etiologies were idiopathic (45.4%) and postinfectious (22.0%). 41.7% had mild bronchiectasis based on the BSI. Patients with bronchiectasis did not differ significantly from healthy controls in demographic characteristics, except for the lower body mass index. The baseline characteristics of 132 patients did not differ from the exacerbation cohort, except for the lower previous exacerbation frequency.

**Table 1:** Baseline characteristics of patients with bronchiectasis and healthy controls.

**Variables**	**Bronchiectasis patients**			**Healthy controls** **(*n* = 50)**	*P*-value^**^
	**Stable visit** **(*n* = 132)**	**Exacerbation visit** **(*n* = 32)**	*P*-value^*^		
**Age (yrs)**	49.0 (40.0–61.0)	53.5 (40.3–63.5)	0.674	43.5 (27.8–56.0)	0.061
**Sex (% females)**	66 (50.0%)	17 (53.1%)	0.751	24 (48.0%)	0.810
**Body–mass index (Kg/m^2^)**	20.8 ± 3.1	20.3 ± 3.2	0.498	23.1 ± 3.5	**<0.001**
**FEV_1_% pred**	58.3 (43.0–75.2)	48.0 (37.4–70.5)	0.058	92.5 (88.0–102.0)	**<0.001**
**FEV_1_/FVC%**	67.3 ± 13.9	64.5 ± 13.4	0.299	84.1 ± 4.8	**<0.001**
**Never–smokers (No., %)**	122 (92.4%)	30 (93.8%)	0.999	50 (100.0%)	0.101
**Exacerbation frequency in the previous year**	1.0 (0.0–1.8)	1.0 (1.0–2.0)	**0.004**	NA	NA
**Etiology**			0.939		NA
Idiopathic (No., %)	60 (45.4%)	16 (50.0%)		NA	NA
Post-infectious (No., %)	29 (22.0%)	7 (21.9%)		NA	NA
Immunodeficiency (No., %)	10 (7.6%)	1 (3.1%)		NA	NA
Post-tuberculous (No., %)	12 (9.1%)	2 (6.2%)		NA	NA
Others (No., %)	21 (15.9)	6 (18.8%)		NA	NA
**Bronchiectasis severity**			0.168		
Mild (No., %)	55 (41.7%)	8 (25.0%)		NA	NA
Moderate (No., %)	42 (31.8%)	15 (46.9%)		NA	NA
Severe (No., %)	35 (26.5%)	9 (28.1%)		NA	NA
Modified Reiff score	10.0 (7.0–14.0)	11 (7.3–15.0)	0.260	NA	NA

*FEV_1_, forced expiratory volume in 1 s; pred%, the percentage predicted; FVC, forced vital capacity; NA, not applicable.*

*Continuous variables were initially checked for normality and expressed as mean ± SD or median (interquartile range) as appropriate. Categorical variables were summarized as count (percentage).*

*The exacerbation column denoted patients with bronchiectasis with an outpatient visit due to having an exacerbation during the longitudinal follow-up.*

*Other etiologies of clinically stable bronchiectasis consisted of primary ciliary dyskineisa (n = 10), asthma (n = 3), diffuse panbronchiolitis (n = 2), gastroesophageal reflux (n = 2), allergic bronchopulmonary aspergillosis (n = 1), congenital lung maldevelopment (n = 1), cystic fibrosis transmembrane conductance regulator-associated disorder (n = 1), and connective tissue disease (n = 1); other etiologies of patients who had an exacerbation consisted of primary ciliary dyskineisa (n = 4), gastroesophageal reflux (n = 1), and connective tissue disease (n = 1).*

*The Bronchiectasis Severity Index was adopted for bronchiectasis severity rating in this table.*

* *p-value for the comparison between patients with clinically stable bronchiectasis and those who had an exacerbation during follow-up.*

** *p-value for the comparison between patients with clinically stable bronchiectasis and healthy controls.*

*Data in bold indicated the statistical comparisons with significance*.

#### Marker Profiling of PBMCs in Cross-Sectional Substudy

**Figure 2:**
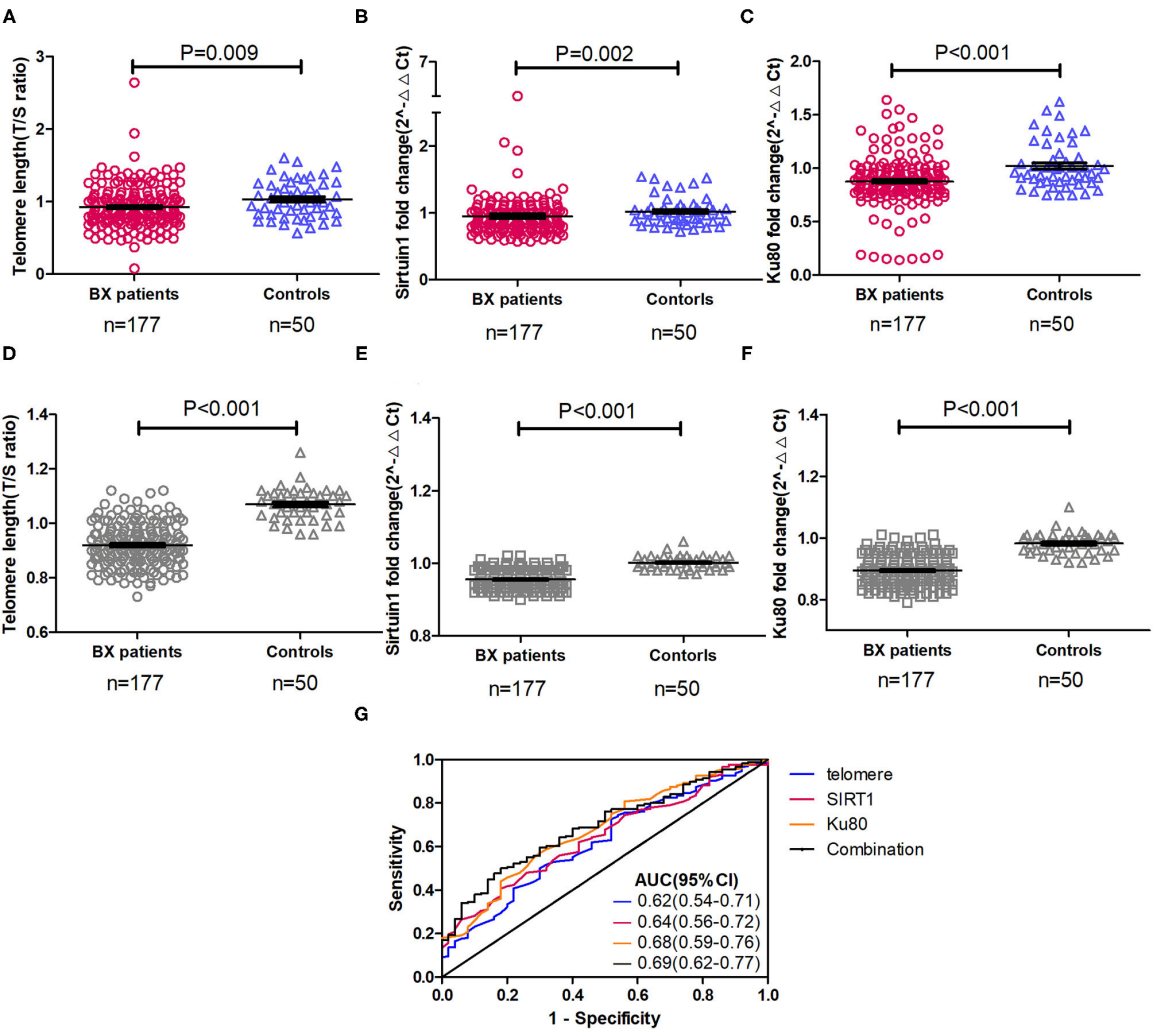
Expression and diagnostic performance of three differentially expressed aging markers in peripheral blood mononuclear cells of patients with clinically stable bronchiectasis and healthy controls. **(A–F)** The expression levels of different aging markers in peripheral blood mononuclear cells of patients with clinically stable bronchiectasis and healthy controls. **(A)** The relative telomere length, expressed as the T/S ratio; **(B)** Sirtuin 1 (SIRT1) expression levels; **(C)** Ku80 expression levels; **(D)** Relative telomere length adjusted with the lung age; **(E)** SIRT1 expression levels adjusted with the lung age; and **(F)** Ku80 expression levels adjusted with the lung age. **(G)** The diagnostic performance of aging markers and their combination to discriminate patients with bronchiectasis from healthy controls. Shown are the three differentially expressed markers between patients with bronchiectasis and healthy controls. The expression level of markers was expressed as the fold change by using the 2^−Δ*Δt*^ cycle threshold algorithm, with exception of the T/S ratio for the relative telomere length. Combination denoted the receiver operating characteristic curve of the sum of the three markers (relative telomere length, SIRT1, and Ku80). A total of 45 patients with bronchiectasis had more than one clinically stable visit, whose data were pooled in the clinically stable visit dataset (*n* = 177). AUC, area under the curve; Bx, bronchiectasis.

Compared with healthy controls, the relative telomere length (median: 0.88 vs. 0.99, *p* = 0.009), SIRT1 (median: 0.89 vs. 0.99, *p* = 0.002), and Ku80 (median: 0.87 vs. 0.96, *p* < 0.001) expression levels were consistently lower in patients with bronchiectasis (Figure 2, Supplementary Table 1). However, there was no between-group difference for other markers. These findings remained valid after adjustment with the lung age (which consisted of age, height, and FEV_1_). Sensitivity analysis that restricted the comparison with the 132 patients with bronchiectasis still demonstrated a significant between-group difference in SIRT1 and Ku80 expression (*p* < 0.05, Supplementary Figure 1). Furthermore, the relative telomere length [AUC: 0.62 (95% CI: 0.54–0.71)], SIRT1 [0.64 (95% CI: 0.56–0.72)], and Ku80 [0.68 (95% CI: 0.59–0.76)] modestly discriminates patients with bronchiectasis from healthy controls. Combining their expression levels, it nominally improved the diagnostic performance [0.69 (95% CI: 0.62–0.77)] (Figure 2). We also analyzed the difference in marker expression in 32 randomly selected patients with bronchiectasis who had dual clinically stable samples (mean interval: 255 days) and the difference for all the markers, except for Ku70 fell within 1.96 times of the SD (Supplementary Figure 2). Both SIRT1 and K80, but not others aging markers, differed among patients with different etiologies (data not shown). There existed no significant sex differences for all the aging markers, except for TERT (91.5% in females vs. 78.0% in males, *p* < 0.05).

**Figure 3:**
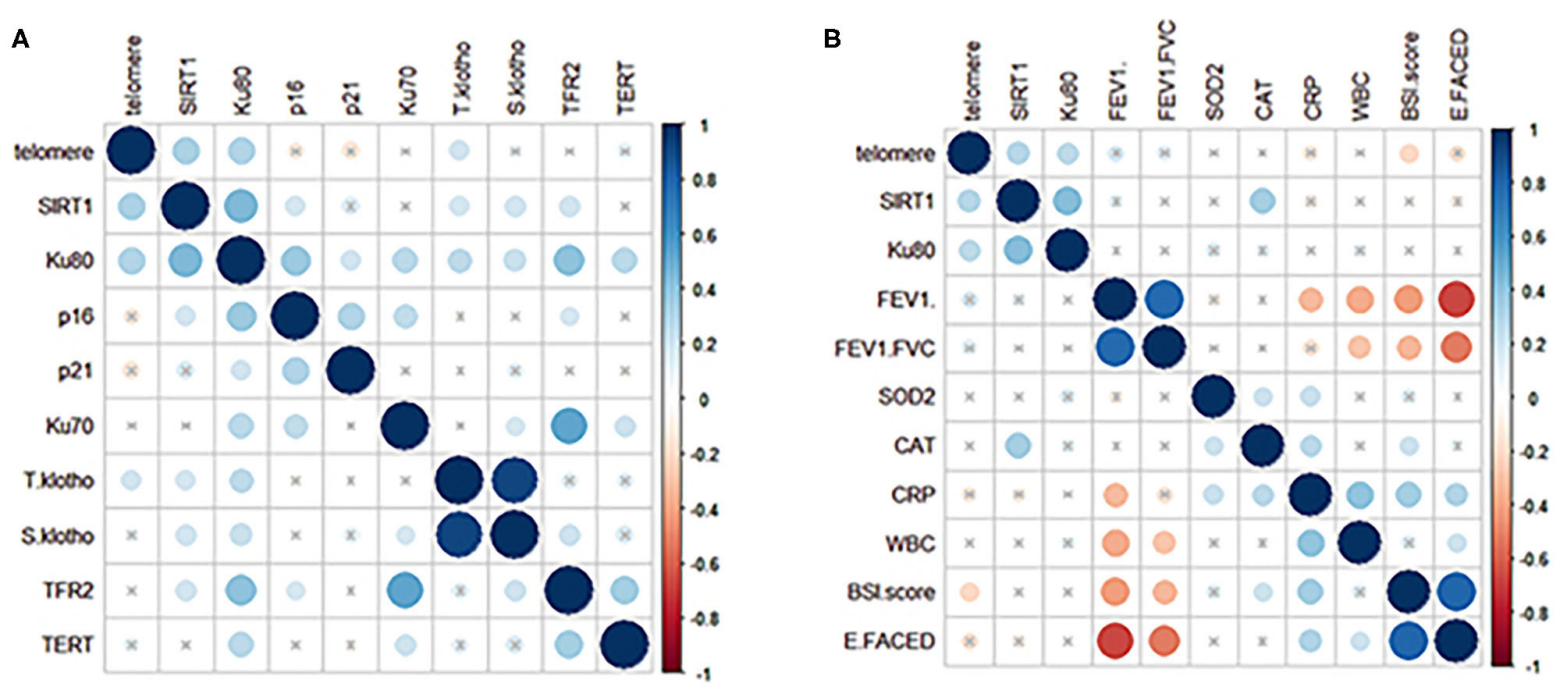
Correlation between the expression levels of aging markers in peripheral blood and the clinical variables in patients with clinically stable bronchiectasis. **(A)** demonstrates the correlation coefficients (shown in different colors reflecting various magnitudes of correlation) for the expression levels of 10 aging markers in clinically stable bronchiectasis. **(B)** displays the correlation coefficients for the association between the clinical variables and the three aging markers, which were differentially expressed between patients with clinically stable bronchiectasis and healthy controls (relative telomere length, SIRT1, and Ku80). Blue dots represent positive correlation whereas red dots reflect negative correlation. Darker colors represent a greater magnitude of correlation. The cross-sign indicates the comparison with no statistical significance.

We next analyzed the correlation among different markers and between aging marker expression and bronchiectasis severity (Figure 3). The strength of correlation was nominally weak-to-modest. The relative telomere length, but not SIRT1 or other markers, correlated with the *BSI* (*r* = −0.162, *p* = 0.031) and the E-FACED score (*r* = −0.172, *p* = 0.023). Stratification of patients into mild-to-moderate and severe bronchiectasis revealed the differential expression in the relative telomere length, Ku70, and TERT (*p* < 0.05), but not SIRT1. SIRT1 failed to discriminate patients with mild-to-moderate from severe bronchiectasis (Supplementary Figures 3, 4). Patients colonized with *Pseudomonas aeruginosa* yielded attenuated expression of TERT (*p* = 0.047), but not with other markers, including SIRT1, than those without SIRT1. Furthermore, we analyzed the association between aging marker expression and individual clinical variables. In Spearman's model, the levels of SIRT1, Ku80, and TERT correlated significantly with the age (Supplementary Table 2). The multivariate linear regression analysis showed that the relative telomere length, but not SIRT1 or Ku80, correlated with FEV_1_ pred% (Table 2).

**Table 2:** The multivariate association between aging marker expression in PBMCs and the clinical variables in bronchiectasis.

**Variable**	**β**	***P*-value**
**Relative telomere length (T/S ratio)**		
Age	**−0.007**	**<0.001**
FEV_1_ pred%	**0.003**	**0.049**
CRP	−0.023	0.260
**The same model with FEV^1^/FVC vs. FEV^1^ pred%**		
FEV_1_/FVC	0.003	0.071
**Sirtuin1 mRNA expression level**		
Age	−0.003	0.198
FEV_1_ pred%	0.001	0.713
Catalase mRNA expression level	**0.373**	**0.018**
**Ku80 mRNA expression level**		
Age	**−0.197**	**0.009**

*FEV_1_, forced expiratory volume in 1 s; pred%, the percentage predicted; CRP, C-reactive protein; FVC, forced vital capacity; PBMCs, peripheral blood mononuclear cells.*

*The Pearson's or Spearman's correlation coefficient was calculated to determine the univariate correlations between aging marker expression levels and the clinical data, after which they were included in the linear multivariate regression analysis, if there was a trend toward significance (p < 0.2).*

*Data in bold indicated the statistical comparisons with significance*.

#### Marker Profiling of PBMCs in Longitudinal Substudy

**Figure 4:**
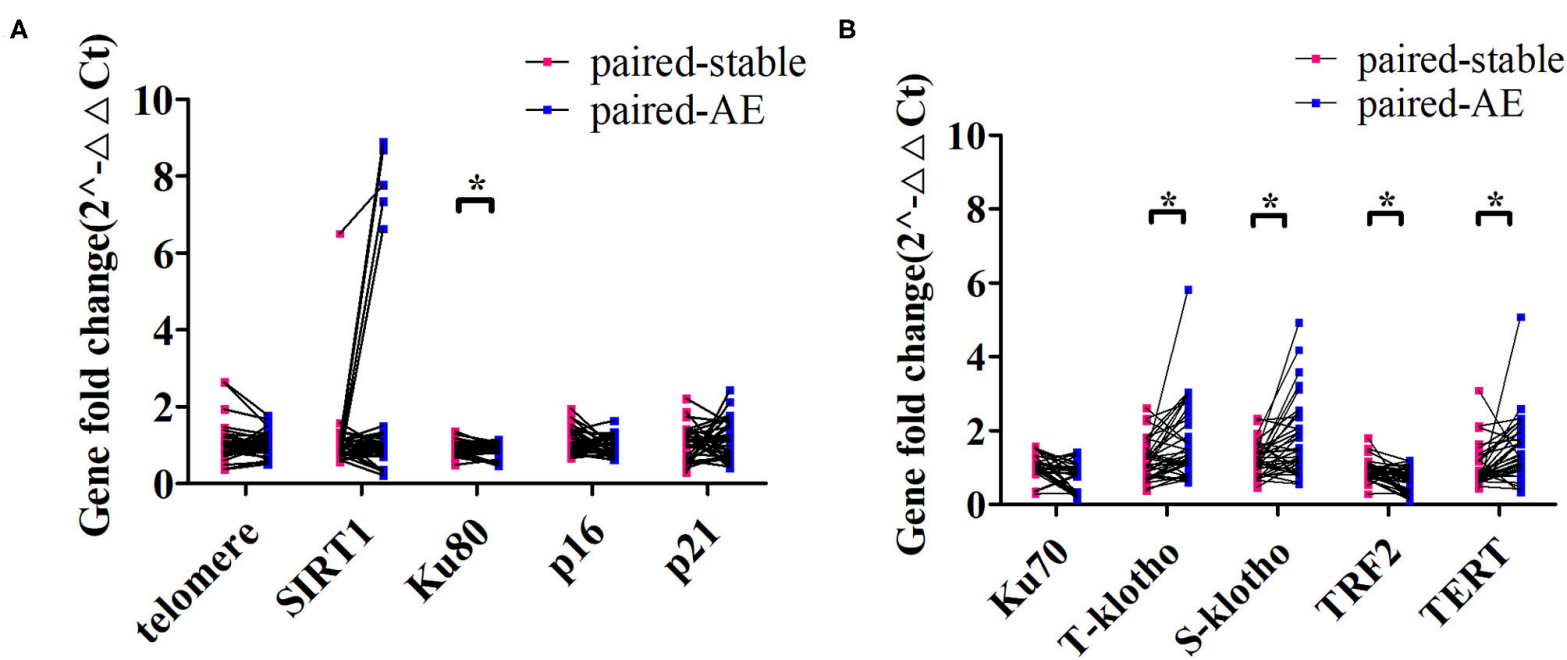
Changes in the expression levels of aging markers from clinically stable to the onset of exacerbation among patients with bronchiectasis. **(A)** demonstrates the comparison of the expression levels of the relative telomere length (telomere), SIRT1, Ku80, p16, and p21 for the paired clinically stable and exacerbation visit samples. **(B)** displays the comparison of the expression levels of Ku70, total klotho (t-klotho), soluble klotho (s-klotho), telomeric repeat-binding factor 2 (TRF2), and telomerase reverse transcriptase (TERT) for the paired clinically stable and exacerbation visit samples. *Denotes the comparisons with statistical significance (*p* < 0.05).

At onset of exacerbation (mean interval from clinically stable visit: 239 days), there was a significant decrease in Ku80 and TRF2 expression and a marked increase in T-klotho, S-klotho, and TERT expression (*p* < 0.05). However, no remarkable changes in SIRT1, telomere length, or Ku70 were identified (Figure 4).

### Study 2

#### Study Participants and Baseline Characteristics

In this cross-sectional substudy, we screened 104 participants between June 2017 and November 2020, 36 of whom were excluded because bronchial epithelium was not identified. Finally, we included 36 patients with bronchiectasis and 32 disease controls (Figure 1B). We sampled the paired large-to-medium and small airway epithelium in 19 patients with bronchiectasis and epithelium specimens from single anatomical location in 17 patients with bronchiectasis (large-to-medium airways in 11 patients and small airways in 6 patients). We also biopsied paired epithelium from 16 disease controls, large-to-medium airways in 6 controls, and small airways only in 10 controls.

The mean age of patients with bronchiectasis was 44.1 years and 52.8% were females. Disease controls were markedly older, although the sex distribution did not markedly differ. The site of biopsy was not significantly different, although sampling of lower lobes favored patients with bronchiectasis (Supplementary Table 3).

#### Aging Marker Profiling of Bronchial Epithelium

**Figure 5:**
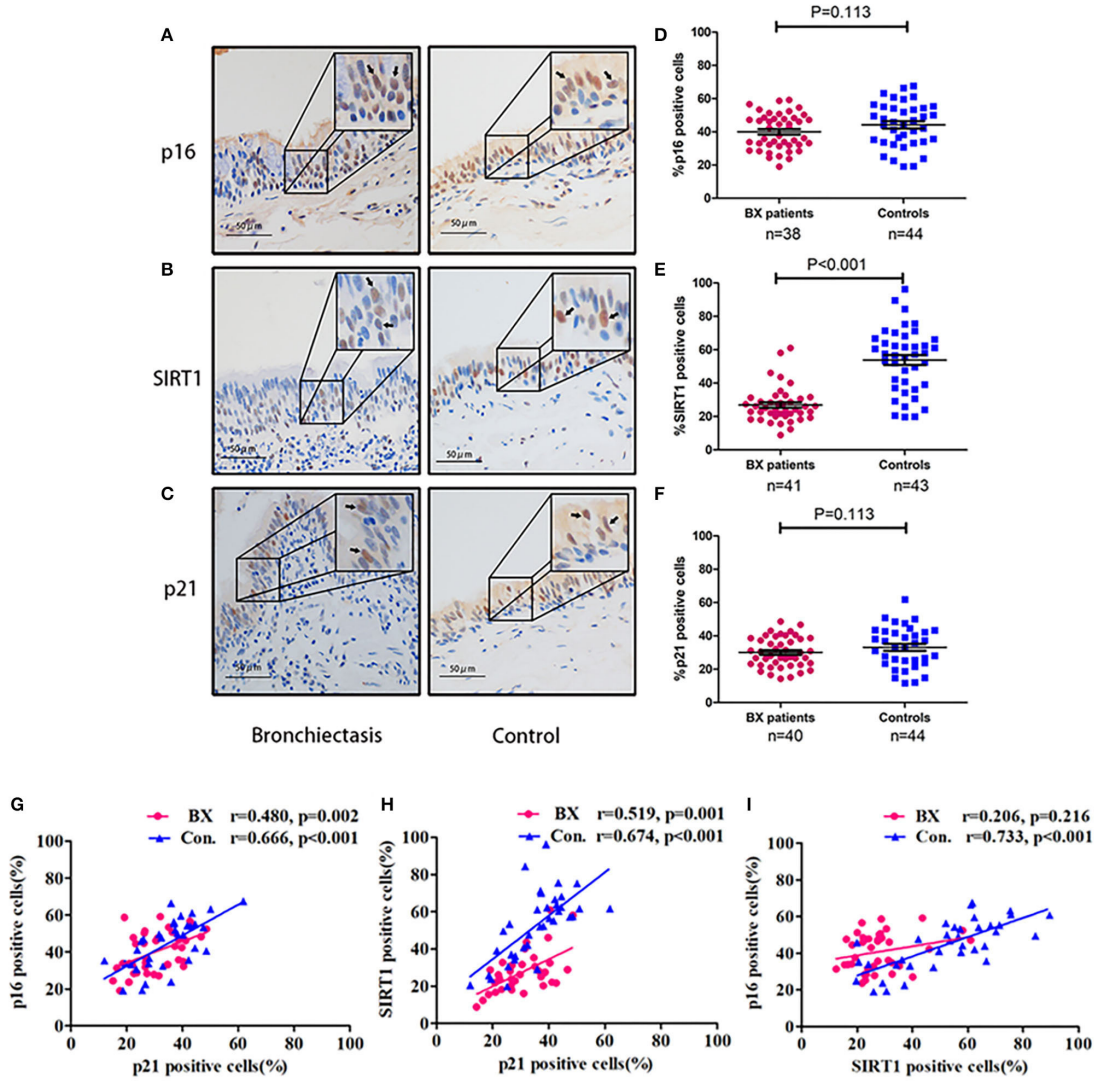
Expression patterns of SIRT1, p16, and p21 within the bronchial epithelium in patients with bronchiectasis and disease controls. **(A–C)** Representative images showing the expression patterns of p16, SIRT1, and p21 within the bronchial epithelium in patients with bronchiectasis and disease controls scheduled for segmentectomy or lobectomy. **(A)** Immunohistochemistry staining of p16; **(B)** Immunohistochemistry staining of SIRT1; and **(C)** Immunohistochemistry staining of p21. The upper right quadrant of each panel demonstrates a magnified image of the positively stained cells, which are indicated with the arrow heads. Positive staining is defined as the presence of staining of the senescence marker within the cell nuclei. **(D–F)** Comparison of the percentage of positively stained cells (vertical axis) for p16, SIRT1, and p21. **(D)** The percentage of cells positively stained with p16; **(E)** The percentage of cells positively stained with SIRT1; and **(F)** The percentage of cells positively stained with p21. **(G–I)** Correlation of the percentage of positively stained cells for p16, SIRT1, and p21; **(G)** Correlation between p16 and p21; **(H)** Correlation between SIRT1 and p21; and **(I)** Correlation between SIRT1 and p16. Bx, bronchiectasis; SIRT1, sirtuin 1; Con, disease control.

**Figure 6:**
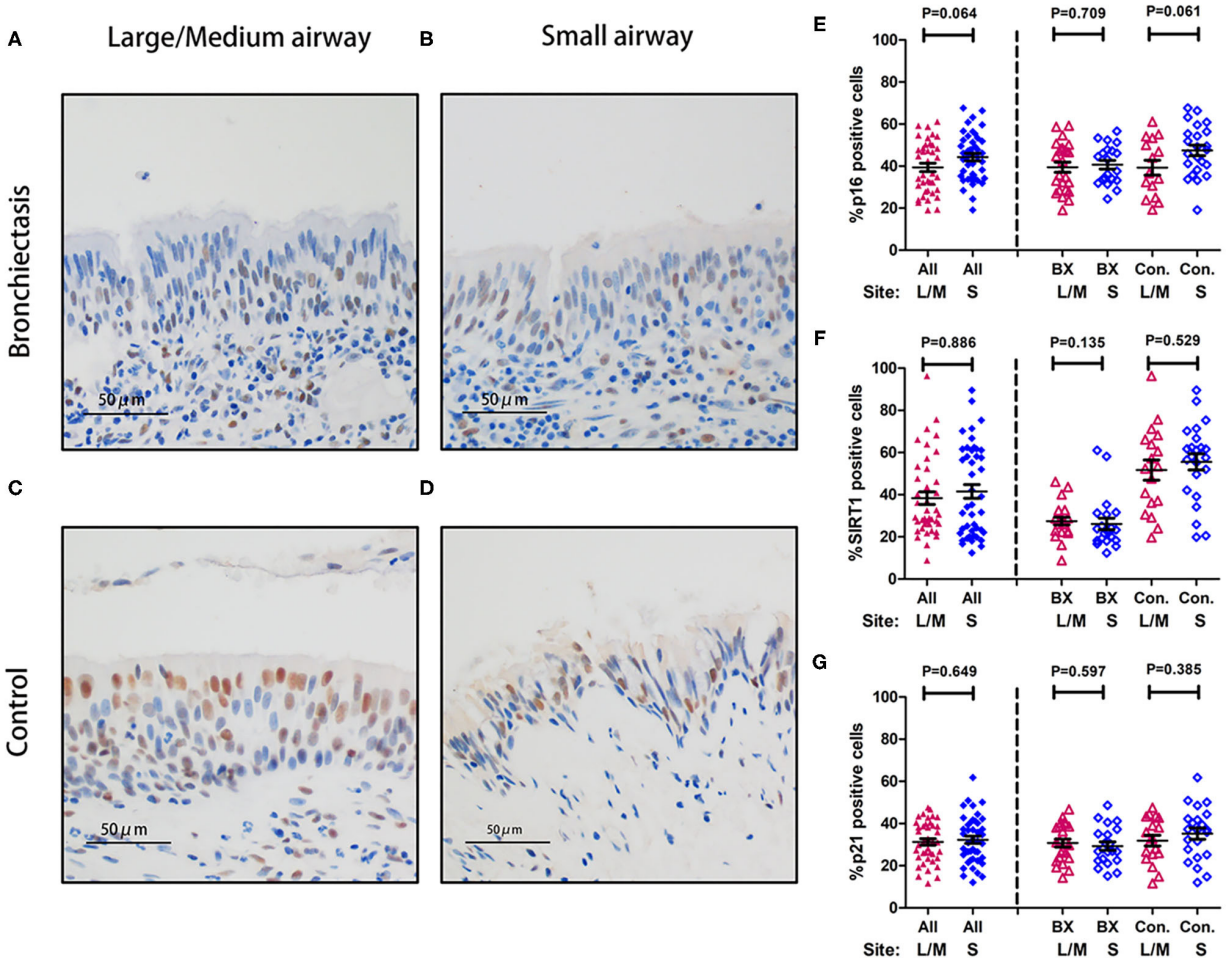
Expression of p16, SIRT1, and p21 between large-to-medium and small airways within the same study participant. **(A–D)** Representative images showing the expression patterns of p16, SIRT1, and p21 within the bronchial epithelium in patients with bronchiectasis and disease controls scheduled for segmentectomy or lobectomy. **(A)** Immunohistochemistry staining within the large-to-medium airway in a patient with bronchiectasis (a 31-year-old female); **(B)** Immunohistochemistry staining within the small airway in a patient with bronchiectasis (a 31-year-old female); **(C)** Immunohistochemistry staining within the large-to-medium airway in a disease control (a 59-year-old male); and **(D)** Immunohistochemistry staining within the small airway in a disease control (a 59-year-old male). Positive staining is defined as the presence of staining of the senescence marker within the cell nuclei. **(E–G)** Dot plots demonstrating the percentage of positively stained cells corresponding to the three senescence markers and different anatomical sites. **(E)** The percentage of positively stained cells for p16; **(F)** The percentage of positively stained cells for SIRT1; and **(G)** The percentage of positively stained cells for p21. All, all study participants; BX, bronchiectasis; Con, disease control; L, large-to-medium airways; S, small airways; SIRT1, sirtuin 1.

Sirtuin 1, p16, and p21 were consistently expressed within cytoplasm and nuclei of the bronchial epithelial cells in both the groups (Figure 5). The percentage of positively stained cells was markedly lower for SIRT1 (median: 25.1 vs. 57.2%, *p* < 0.05) and nominally lower for p16 (median: 40.0 vs. 45.1%) and p21 (median: 28.9 vs. 35.9%) in patients with bronchiectasis than in disease controls (*p* > 0.05) (Supplementary Table 4). We also compared the percentage of positively stained cells for each marker, revealing no significant differences between the large-to-medium airways and small airways in both the groups (Figure 6).

The percentage of positively stained cells for SIRT1 correlated positively with that of p16 and p21 (*p* > 0.05, Figure 5), but not between SIRT1 and p16 in patients with bronchiectasis. Furthermore, the percentage of positively stained cells for SIRT1 correlated weakly with age (*r* = 0.231, *p* = 0.035) and FEV_1_ pred% (*r* = 0.294, *p* = 0.014). There was also a weak correlation between the percentage of positively stained cells for p21 and FEV_1_ pred% (*r* = 0.312, *p* = 0.011) (Supplementary Table 5).

## Discussion

Sirtuin 1 plays a key role in modulating the magnitude of ageing. This cross-sectional substudy showed that patients with bronchiectasis yielded a downregulation of SIRT1 in both the systemic circulation and different sites of bronchial epithelium in bronchiectasis, which remained valid after adjustment with the lung age. SIRT1 expression in the systemic circulation correlated with neither lung function impairment nor the integrated severity metrics. Moreover, the longitudinal substudy also showed that SIRT1 expression did not vary at exacerbation onset.

Accelerated aging has been increasingly recognized responsible for the defective cell repair, and hence, altered microenvironment in chronic airway diseases. Signals reflecting accelerated aging have been detected in PBMCs and airway epithelium among patients with COPD and IPF (6, 20–22). A pilot study has revealed reduced SIRT1 expression in the bronchiectatic airways (8). In this study, we profiled SIRT1 and other aging marker in both the PBMCs and bronchiectatic epithelium. Aging marker profiling in PBMCs has been validated in COPD (14). For instance, there was a good correlation between the telomere length derived from PBMCs and that from lung tissues (23). Therefore, PBMCs served as a proxy of aging within lung tissues.

Sirtuins are a family of deacetylase, which have phototropic effects. SIRT1 reflected the antiaging capacity and longevity (24). This findings partly echoed those by Rutten and colleagues, who demonstrated reduced SIRT1 expression in COPD, which correlated positively with the expression of catalase (14). This might be because oxidative stress attenuated SIRT1 expression via activating the phosphatidylinositol 3Kα signaling pathway (25). Indeed, antioxidants had a role in modulating the lifespan in several animal models such as *Caenorhabditis elegans* (26). However, this findings did not justify the correlation between SIRT1 expression and bronchiectasis severity. By comparison, SIRT1 expression in PBMCs did not differ between mild asthma and severe asthma (27) and SIRT1 expression in both the sputum supernatant and serum did not correlate with asthma severity (28). The *BSI* and the E-FACED score consisted of several domains such as exacerbation frequency and bacterial colonization, which did not correlate with SIRT1 expression independently. These indicated the dissociation between SIRT1 expression and bronchiectasis severity.

Overall, the correlation between SIRT1 and other marker expression was weak to modest. The other two markers that correlated positively with SIRT1 consisted of the relative telomere length and Ku80. The relative telomere length and Ku80 were differentially expressed in PBMCs and correlated with bronchiectasis severity, which echoed the findings in patients with COPD (29). These markers reflected different pathways modulating cell aging. However, we did not identify differential expression of other markers within PBMCs in bronchiectasis. These findings differed from the observations in COPD, possibly reflecting distinct pathophysiology of accelerated aging.

The variation in aging marker expression was minimal when clinically stable and became significant at onset of exacerbation compared with baseline levels. These notable changes might have been associated with the aggravated infections or inflammatory responses. Further studies are needed to verify these observations.

We have revealed markedly reduced SIRT1 but not p16 or p21 in bronchial epithelium, which partially differed from the findings of reduced SIRT1 expression but higher p21 expression in patients with bronchiectasis (8). The differences in the control population (IPF or lung tumors vs. healthy controls) might have contributed to the disparity because IPF (especially patients scheduled for lung transplantation) and lung tumors have been associated with accelerated aging (11), although we have made efforts to minimize the impact by sampling epithelium at least 1 cm apart from the tumor lesions. Moreover, the reduced SIRT1 expression was not tempered significantly by the inclusion of disease controls and the markedly greater age in patients with bronchiectasis. Reassuringly, aging marker expression in large-to-medium and small airways was comparable, which would help to minimize the need to sample distal airways which cannot be readily derived with conventional biopsy techniques.

Studies of the aging marker expression have been performed in many chronic respiratory diseases such as COPD and asthma. The published study documented a reduction in SIRT1 expression in bronchiectasis, but the association with the clinical characteristics has not been outlined. In this study, we have not only profiled the expression level of a panel of aging markers associated with different pathways, but also demonstrated the diagnostic value, the correlation with important clinical metrics (e.g., BSI, HRCT score, and lung function), and the changes at onset of exacerbation as compared with the baseline levels. Furthermore, we have also shown the significant correlation of some, but not all of the aging marker expression levels. These findings have provided further insights into the role of accelerated aging in bronchiectasis.

This findings might have some clinical implications. SIRT1 expression could be measured in adults with bronchiectasis as a marker of accelerated aging. The decreased SIRT1 expression might be clinically relevant to therapeutic interventions because resveratrol could ameliorate oxidative stress and inflammation via activating SIRT1 in murine COPD models (30). It would be interesting to ascertain whether measures that aim to counter the accelerated aging might have a role in improving the clinical outcomes of bronchiectasis. Furthermore, because of the difficulty in directly biopsying small airways, assessment of aging could either be achieved via isolating PBMCs or sampling large-to-medium airways.

This data interpretation was constrained by the limited PBMCs samples captured at exacerbation. Outpatient services have been suspended during the outbreak of coronavirus disease 2019 and because of the policy to mitigate nosocomial cross-contamination, the follow-up scheme has been withheld between January and June 2020. We cannot comment on the impact of smoking on aging. However, the small number of ever-smokers suggested minor confounding effects. One of our important goals was to determine whether the expression levels of aging markers would differ dramatically in different portions of the tracheobronchial tree. Therefore, we have biopsied the surgically excised epithelium among patients who were scheduled for lung resection and transplantation instead of sampling via bronchoscopy. Hence, sampling from the completely healthy controls or patients with peripheral lung nodules who underwent bronchoscopy would not be possible. In this study, patients with IPF or lung tumors have constituted the disease controls, which could have partly obscured the difference in p16 and p21 expressions. In spite of this, the markedly reduced SIRT1 expression in patients with bronchiectasis as compared with the disease controls suggested that this findings pertaining to the role of SIRT1 in mediating accelerated aging in bronchiectasis remained valid. We did not enroll age-matched patients with bronchiectasis in Study 2, which might have confounded the expression levels of aging markers. However, the greater age in the disease controls would have maximized rather than diluted the between-group difference in the percentage of cells staining positive for the aging markers, which was clearly not the case in this study. In spite of these limitations, this study demonstrated lower percentage of cells staining positive for SIRT1 in patients with bronchiectasis, suggesting that the reduction in SIRT1 expression would have been more prominent. We did not analyze the consistency of aging marker expression levels in the PBMCs and bronchial epithelium derived from the same individual patients because the paired blood samples were not collected for this study in patients undergoing surgical resections. Although SIRT1 expression was independent of the disease severity and lung function impairment in this cohort, it would be vital and reasonable to assess the relationship between aging marker expression levels and clinically important outcomes such as mortality in future large-scale longitudinal studies given the lack of objective surrogate markers for survival prediction in patients with bronchiectasis at present. Validation of this findings in other patient populations is also needed. Finally, not all the markers were validated in the bronchial epithelium study and we selected three markers given their relationship with the aging pathways and the technical feasibility of staining.

In conclusion, we have unraveled the marked reduction in SIRT1 expression in both the systemic circulation and bronchiectatic airways, which is independent of lung function impairment. Further studies are needed to provide mechanistic insights into the functional implications of these findings.

## Data Availability Statement

The original contributions presented in the study are included in the article/Supplementary Materials, further inquiries can be directed to the corresponding author/s.

## Ethics Statement

The Ethics Committee of the First Affiliated Hospital of Guangzhou Medical University (Medical Ethics 2016, the 32th) approved for the study protocol. All participants gave written informed consent. The patients/participants provided their written informed consent to participate in this study.

## Author Contributions

X-rH, W-jG, and Y-hG participated in study design and drafted the manuscript. X-rH, H-mL, L-jC, C-xP, Z-hL, R-lZ, and YH performed laboratory experiments and data analysis. X-rH, H-mL, L-jC, C-xP, Z-hL, R-lZ, YH, and W-jG recruited patients and performed follow-up. W-jG and Y-hG provided critical review of the manuscript. All authors have approved the final draft of the manuscript for publication.

## Funding

This study was supported by the National Natural Science Foundation (No. 81870003) and the Zhongnanshan Medical Foundation of Guangdong Province (No. ZNSA-2020013) (to W-jG) and Shanghai Pujiang Program 2021 (21PJD061) (to Y-hG).

## Conflict of Interest

The authors declare that the research was conducted in the absence of any commercial or financial relationships that could be construed as a potential conflict of interest.

## Publisher's Note

All claims expressed in this article are solely those of the authors and do not necessarily represent those of their affiliated organizations, or those of the publisher, the editors and the reviewers. Any product that may be evaluated in this article, or claim that may be made by its manufacturer, is not guaranteed or endorsed by the publisher.
