# Supplementary figures and images for: Decreased Systemic and Airway Sirtuin 1 Expression in Adults With Bronchiectasis
Source: Front Med (Lausanne). 2022 Jan 6;8:768770. doi: 10.3389/fmed.2021.768770 (PMC8770945; doi:10.3389/fmed.2021.768770)

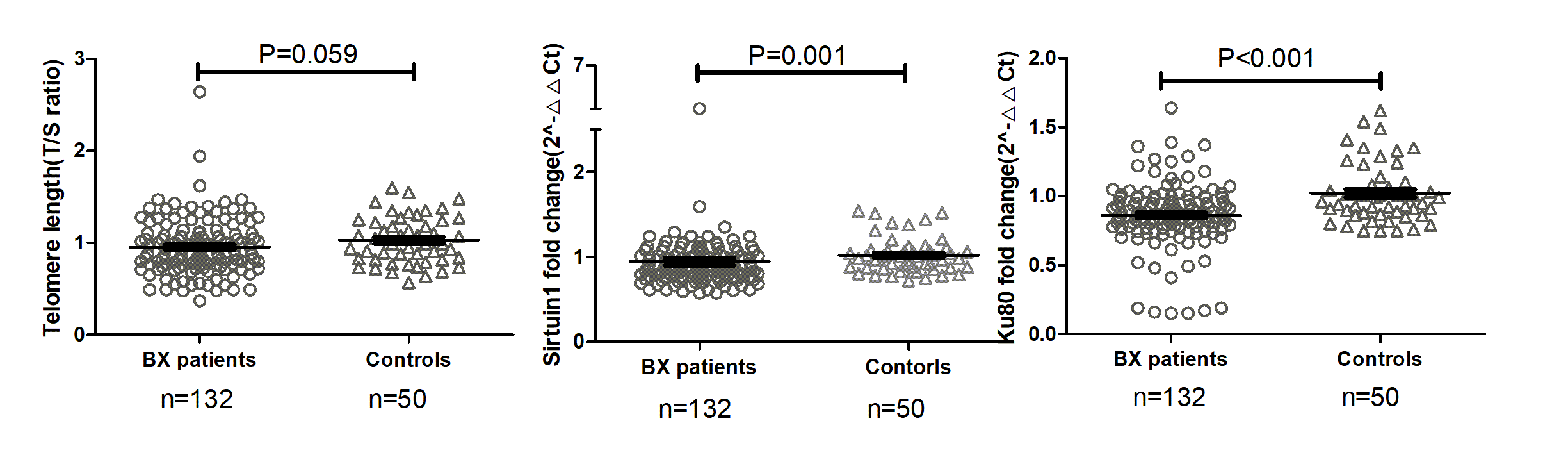

Supplement: Supplementary file 1 [file Image_1.TIF]

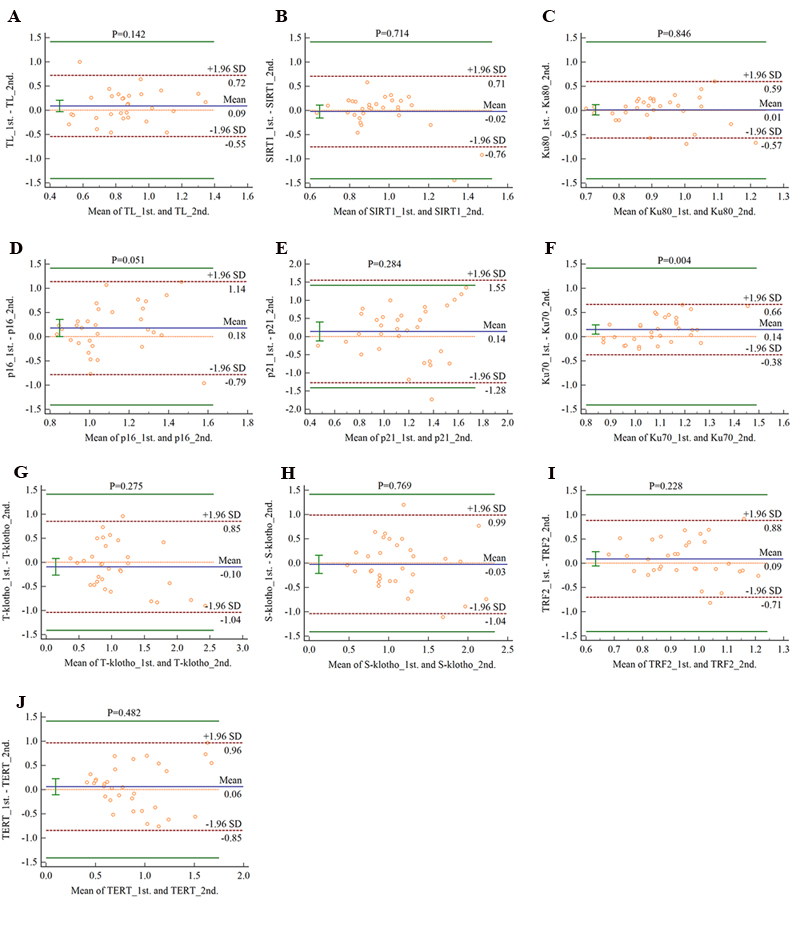

Supplement: Supplementary file 2 [file Image_2.JPEG]

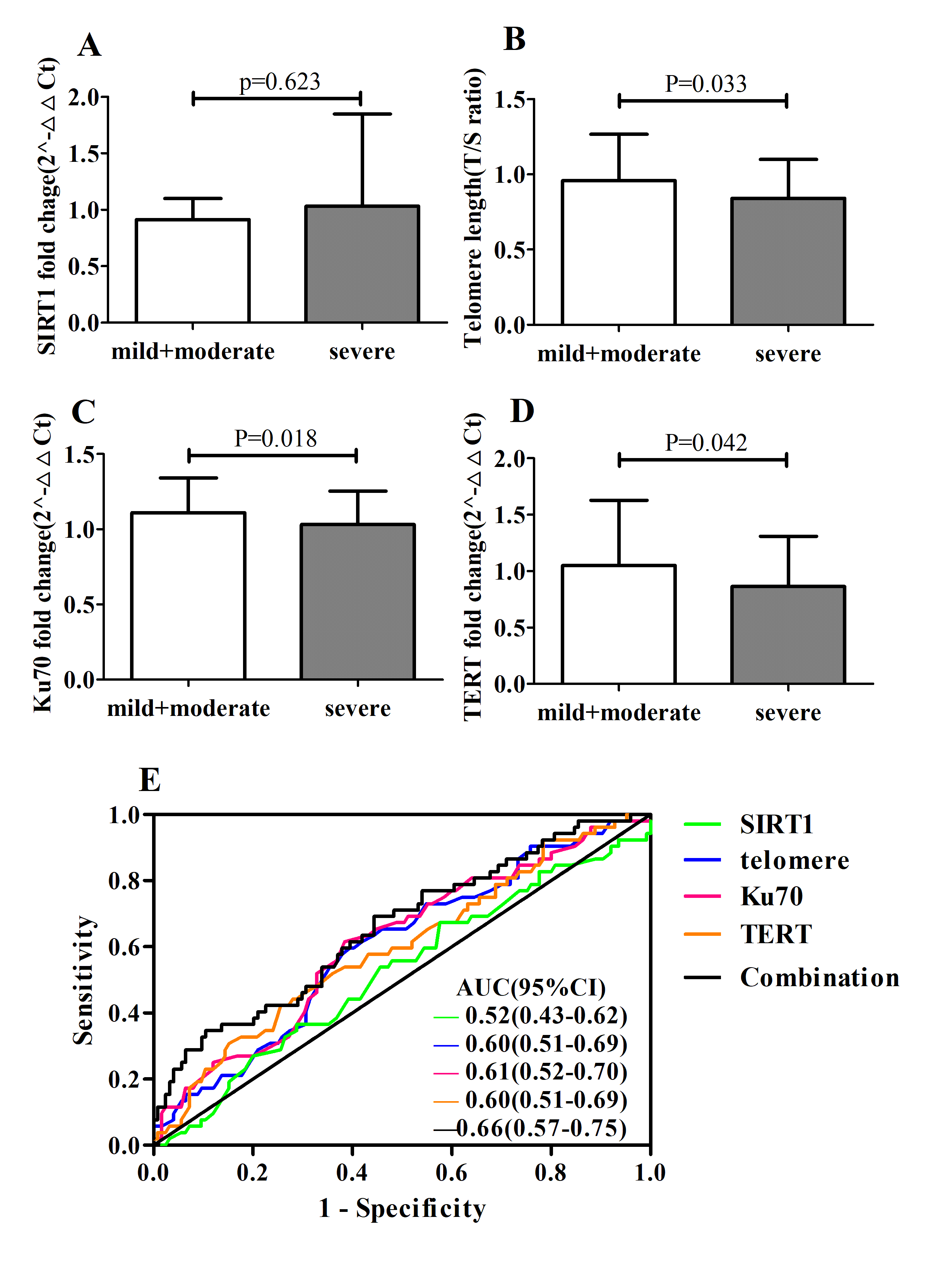

Supplement: Supplementary file 3 [file Image_3.TIF]

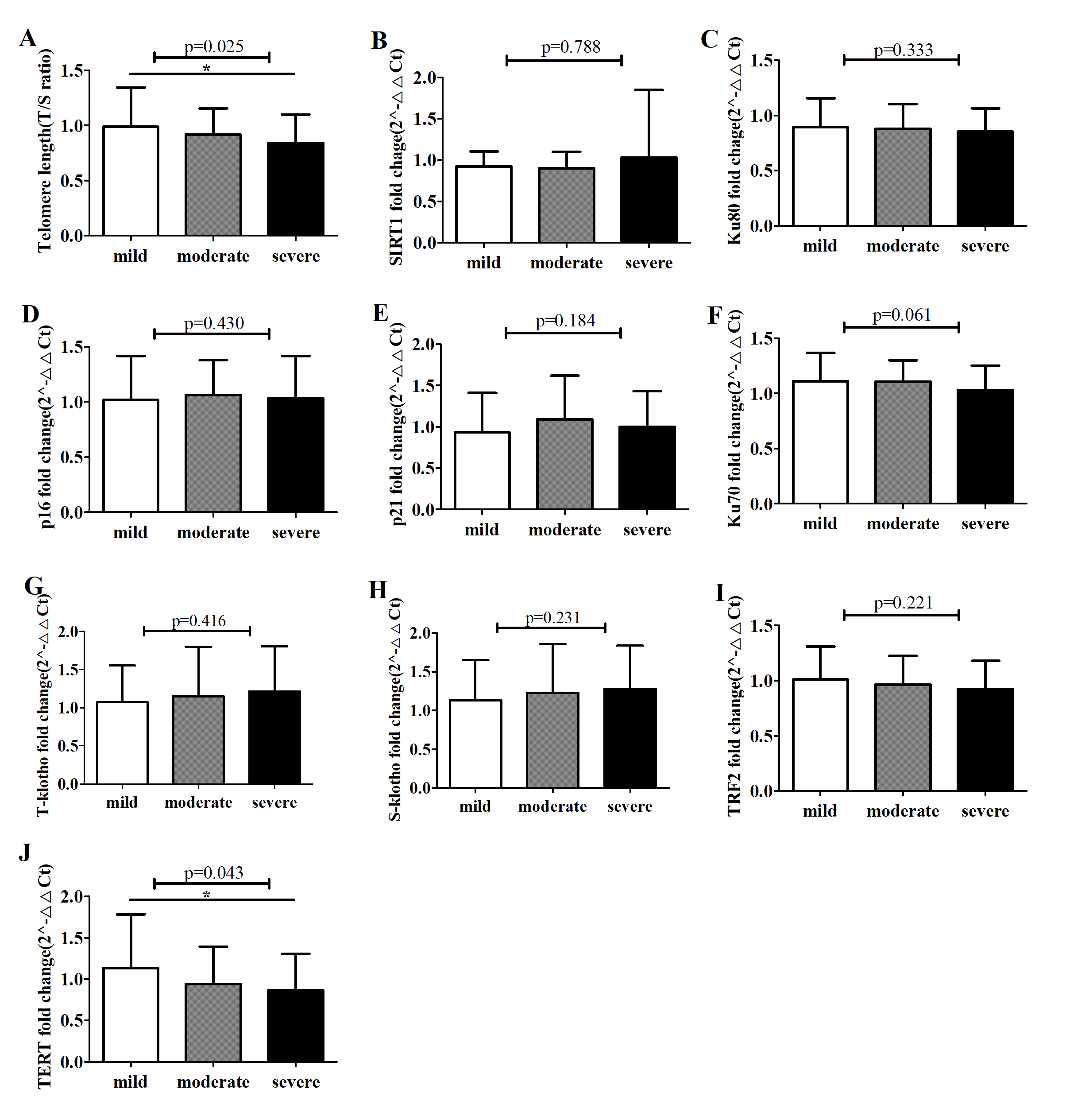

Supplement: Supplementary file 4 [file Image_4.TIF]
