# Supplementary material for: Decreased Systemic and Airway Sirtuin 1 Expression in Adults With Bronchiectasis
Source: Front Med (Lausanne). 2022 Jan 6;8:768770. doi: 10.3389/fmed.2021.768770 (PMC8770945; doi:10.3389/fmed.2021.768770)
Supplement: Supplementary file 5 [file Table_2.DOCX]

**Online supplement**

**Decreased systemic and airway sirtuin 1 expression in adults with bronchiectasis**

**Xiao-rong Han 1, Lai-jian Cen 1, Cui-xia Pan 1, Zhen-hong Lin 1, Hui-min Li 1, Ri-lan Zhang 1, Yan Huang 1, Yong-hua Gao 2, Wei-jie Guan 1,3**

^1^ State Key laboratory of Respiratory Disease, National Clinical Research Center for Respiratory Disease, Guangzhou Institute of Respiratory Health, The First Affiliated Hospital of Guangzhou Medical University, Guangzhou, China

^2^ Shanghai Pulmonary Hospital, Shanghai, China

^3^ Department of Thoracic Surgery, Guangzhou Institute of Respiratory Disease, First Affiliated Hospital of Guangzhou Medical University, Guangzhou, Guangdong, China

**Corresponding author:** Wei-Jie Guan; State Key Laboratory of Respiratory Disease, National Clinical Research Center for Respiratory Disease, Guangzhou Institute of Respiratory Health, The First Affiliated Hospital of Guangzhou Medical University, 151 Yanjiang Road, Guangzhou, Guangdong, China. Tel: +86-20-83062876; Fax: +86-20-83062719; E-mail: [battery203@163.com](mailto:battery203@163.com)

Yong-hua Gao; Department of Respiratory and Critical Care Medicine, Shanghai Pulmonary Hospital, Tongji University School of Medicine, Shanghai, China. Address: No. 507 Zhengmin Road, Shanghai, 200433, China; Tel: +86-15938709683; E-mail: [gaoyonghuahust@163.com](mailto:gaoyonghuahust@163.com)

**Methods**

***Study participants***

Study 1

We recruited study participants aged 18 to 75 years between June 2017 and January 2021. To profile senescence markers in PBMCs, we included symptomatic (i.e., cough, sputum production) patients with bronchiectasis, and the diagnosis was based on chest high-resolution computed tomography, effective within 12 months. Clinical stability denoted the magnitude of symptom burden (i.e., cough, sputum production) that did not significantly exceed the normal daily variations. All patients were free from the use of any nebulized, oral, or intravenous antibiotics (except for the maintenance low-dose macrolides) within 4 weeks prior to the study entry.

For clinical stability visits, we excluded the bronchiectasis patients who had an exacerbation or antibiotic use within 4 weeks. We recruited healthy controls who had normal chest X-ray and spirometry, and were free from lower airway symptoms or severe systemic diseases. We excluded participants who were pregnant or breastfeeding, and those who had limited understandings.

We also collected the PBMCs among a subgroup of bronchiectasis patients during the onset of exacerbations at longitudinal follow-up. In our study, the diagnosis of exacerbation was made based on the 2017 *European Respiratory Society* expert consensus criteria. Briefly, an exacerbation was defined as the significant worsening of at least three of the following symptoms which persisted for more than 48 hrs and needing a change in the current therapeutic strategy: cough, sputum volume or consistency, sputum purulence, dyspnea or reduced exercise capacity, fatigue or malaise, hemoptysis.

Study 2

To characterize ageing marker expression in bronchial epithelium, we enrolled patients with bronchiectasis patients (bronchiectasis group), and those with idiopathic pulmonary fibrosis (IPF) or lung tumor scheduled for segmentectomy or lobectomy (disease controls).

We evaluated the bronchial epithelium from 36 bronchiectasis patients and 32 disease controls subjects [including the 21 adjacent normal tissue from subjects with tumor (19 with pulmonary invasive adenocarcinoma, 1 with pulmonary hamartoma, 1 with atypical adenomatous hyperplasia) and 11 with idiopathic pulmonary fibrosis].

Typically, the surgically resected bronchi corresponded to the airways that were most severely affected. In disease controls, the presence of bronchiectasis was excluded based on the radiologic manifestations on the chest HRCT. For disease controls who had tumor, the bronchial epithelium at least 1 cm away from the tumor’s border was sampled to minimize the impact on senescence marker expression.

The study protocol was approved by the ethics committee of The First Affiliated Hospital of Guangzhou Medical University (Medical Ethics 2016, the 32^th^). All participants gave written informed consent.

**HRCT rating**

The diagnosis and classification of bronchiectasis was made according to the chest high-resolution computed tomography (HRCT). The HRCT scores (Modified Reiff Score) were recorded to evaluate the severity of bronchial dilatation (tubular: 1 point, varicose: 2 points, cystic: 3 points), with the maximal score of 18 for six lung lobes.

**Isolation of peripheral blood mononuclear cells**

The fresh peripheral blood was collected into an anticoagulant tube and processed within 12 hours, followed by gentle mixture. Next, the whole blood was diluted with an aliquot of phosphate buffer solution, and mixed with an aliquot of the human leukocyte separation medium (Dakewe Inc., Shenzhen, China), which sat for the separation of the different liquid layers for centrifugation at 800g for 25 min. The buffy coat that contained PBMCs was meticulously aspirated from the interface of the upper low layers (isolation medium vs. plasma layer) for storage in -80 degree freezers before analysis.

The DNA was extracted with the commercial extraction kit (Takara Inc., Osaka, Japan) for assessment of the relative leukocyte telomere length (T/S ratio). The RNA was extracted with Trizol reagent and reverse transcribed for profiling of the senescence markers.

**Systems of the reverse transcription**

**Step 1. Mixtures for removal of the genomic DNA**

| **Reagents** | **Volume (μl)** |
| --- | --- |
| 5x gDNA Eraser Buffer | 2.0 |
| gDNA Eraser | 1.0 |
| Total RNA | 4.0 |
| RNase Free dH_2_O | 3.0 |

The assay was performed at 42℃ for 2 min, followed by water bath at 4℃.

**Step 2. Reverse transcription reactions**

| **Reagents** | **Volume (μl)** |
| --- | --- |
| The mixture mentioned above | 10.0 |
| PrimeScript RT Enzyme Mix I | 1.0 |
| RT Primer Mix | 1.0 |
| 5×PrimeScript Buffer 2 (for Real Time) | 4.0 |
| RNase Free dH_2_O | 4.0 |
| **Total** | **20.0** |

The assay was conducted at 37 ℃ for 2 min and 85 ℃ for 5 sec, followed by water bath at 4 ℃.

**Detection of mRNA expression levels of the senescence markers in the PBMCs**

The primers are listed below.

**Table A. Primers for the RT-PCR assays**

| **Genes** | **Forward primers (5’-3’)** | **Reverse primers (5’-3’)** |
| --- | --- | --- |
| Sirtuin1 | AAGTTGACTGTGAAGCTGTACG | TGCTACTGGTCTTACTTTGAGGG |
| p16 | GGGTTTTCGTGGTTCACATCC | CTAGACGCTGGCTCCTCAGTA |
| p21 | TGTCCGTCAGAACCCATGC | AAAGTCGAAGTTCCATCGCTC |
| Ku70 | AATGACAGTGCCAAAGCCAGC | CAAGGATATGTCAAAGCCCCC |
| Ku80 | GCACTGACAATCCCCTTTCTG | TCAATGTCCTCCAGCAAATCAAA |
| TRF2 | TGACAGAAGCAGTGGTCGAA | CAGCTTCTGAGTTGTGGGGT |
| T-klotho | CCCTAAGCTCTCACTGGATCA | GGCAAACCAACCTAGTACAAAGT |
| S-klotho | GGCTTCCCTCCTTTACCTGAAA | GAGACTGCTGATTGGTTTTGTCA |
| TERT | CGGAAGAGTGTCTGGAGCAA | GGATGAAGCGGAGTCTGGA |
| SOD2 | CGTCACCGAGGAGAAGTACC | TGACCACCACCATTGAACTT |
| Catalase | AGATGCAGCACTGGAAGGAG | ACGGGGCCCTACTGTAATAA |
| HPRT1 | CCTGGCGTCGTGATTAGTGAT | AGACGTTCAGTCCTGTCCATAA |

Reaction system for the RT-PCR

| **Reagents** | **Volume (μl)** |
| --- | --- |
| TB Green Premix Ex Taq Ⅱ (Tli RNaseH Plus) (2x) | 10 |
| PCR Forward Primer (10μM) | 0.8 |
| PCR Reverse Primer (10μM) | 0.8 |
| ROX Reference Dye (50x) | 0.4 |
| DNA templates | 2 |
| Sterilized water | 6 |
| Total | 20 |

Denaturing was set at 95℃ for 30 sec, followed by extension at (95℃ for 5 sec + 60℃ for 34 sec) × 40 cycles.

**Measurement of the relative telomere length**

**Table B. Primer sequences for measuring the relative telomere length**

|  | **Forward primer (5'--3')** |
| --- | --- |
| **telomere-F** | ACACTAAGGTTTGGGTTTGGGTTTGGGTTTGGGTTAGTGT |
| **telomere-R** | TGTTAGGTATCCCTATCCCTATCCCTATCCCTATCCCTAACA |
| **albumin-F** | CGGCGGCGGGCGGCGCGGGCTGGGCGGAAATGCTGCACAGAATCCTTG |
| **albumin-R** | GCCCGGCCCGCCGCGCCCGTCCCGCCGGAAAAGCATGGTCGCCTGTT |

**Reaction system for the relative telomere length**

**Table C. Reaction system for telomere and albumin**

| **Reagents** | **Volume (μl)** |
| --- | --- |
| TB Green Premix Ex Taq Ⅱ(Tli RNaseH Plus)(2x) | 10.0 |
| Telomere Forward Primer (10μM) | 0.3 |
| Telomere Reverse Primer (10μM) | 0.3 |
| ROX Reference Dye (50x) | 0.3 |
| DNA template | 2.0 |
| Sterilized water | 2.1 |
| Total | 15.0 |

**Conditions:**

Stage 1: 95℃ for 15 min;

Stage 2: (94℃ for 15 sec + 49℃ for 15 sec) × 2 cycles;

Stage 3: (94℃ for 15 sec + 62℃ for 10 sec + 73℃ for 15 sec) × 25 cycles

| **Reagents** | **Volume (μl)** |
| --- | --- |
| TB Green Premix Ex Taq Ⅱ(Tli RNaseH Plus)(2x) | 10.0 |
| Albumin Forward Primer (10μM) | 1.35 |
| Albumin Reverse Primer (10μM) | 1.35 |
| ROX Reference Dye (50x) | 0.3 |
| DNA template | 2.0 |
| Total | 15.0 |

**Internal control - Albumin**

**Conditions:**

Stage 1: 95℃ for 15 min;

Stage 2: (94℃ for 15 sec + 49℃ for 15 sec) × 2 cycles;

Stage 3: (94℃ for 15 sec + 62℃ for 10 sec + 88℃ for 15 sec) × 33 cycles

***Tissue preparation and immunohistochemistry staining***

We fixed the surgically resected bronchial epithelium in 4% paraformaldehyde, and embedded in paraffin. Next, we dewaxed three-micrometer thick tissue sections in xylene, rehydrated with graded ethanol solutions, washed in distilled water and subject to heat-induced antigen retrieval in Tris–ethylene diamine tetra-acetic acid buffer at 95℃ for 15 min and cooled at room temperature. We permeabilized the sections with 0.2% Triton X-100 in phosphate buffer solution, blocked with 10% goat-serum and incubated with primary polyclonal antibodies of p21 (1:100, anti-p21, Abcam, USA), p16 (1:100, anti-p16, Abcam, USA) and SIRT1 (1:200, anti-SIRT1, Abcam, USA). We washed the slides thrice with Tris buffer solution, incubated with diaminobezidin (1:50, DAKO A/S, Denmark) at 37℃, and washed thrice in Tris buffer solution. We processed negative controls without the primary antibody. We then counterstained the slides with hematoxylin.

We obtained images at ×400 magnifications with the light microscopy (Olympus ckx53, Osaka, Japan). We randomly evaluated five high-power fields (by two independent reviewers blinded to patient’s profiles), through calculating the percentage of positively stained cells (with the cell nuclei expressing the marker). We then averaged the mean reading of five fields. We adjudicated by means of consensus for any major disagreement (>10% difference) in the cell counts between two reviewers.

**Results**

**Table E1. The expression levels of senescence markers and the oxidative stress genes between bronchiectasis patients and healthy controls**

|  | **Median (interquartile range)** | |  |
| --- | --- | --- | --- |
| **Markers** | **Bronchiectasis patients (n=177)** | **Healthy controls (n=50)** | **P value** |
| **Relative telomere length (T/S ratio)** | 0.88 (0.73-1.07) | 0.99 (0.82-1.24) | **0.009** |
| **Telomerase mRNA levels** |  |  |  |
| **TERT** | 0.82 (0.65-1.23) | 0.99 (0.73-1.33) | 0.144 |
| **Anti-senescence markers mRNA levels** |  |  |  |
| Sirtuin1 | 0.89 (0.77-1.02) | 0.99 (0.87-1.09) | **0.002** |
| T-klotho | 0.99(0.72-1.32) | 0.97 (0.72-1.54) | 0.997 |
| S-klotho | 1.11 (0.79-1.44) | 0.84 (0.73-1.52) | 0.198 |
| **Cell senescence markers mRNA levels** |  |  |  |
| p16 | 0.98 (0.82-1.23) | 1.03 (0.83-1.19) | 0.841 |
| p21 | 0.97 (0.63-1.29) | 1.02 (0.70-1.40) | 0.317 |
| **DNA repair markers mRNA levels** |  |  |  |
| TRF2 | 0.93 (0.78-1.12) | 0.98 (0.86-1.31) | 0.098 |
| Ku70 | 1.04 (0.94-1.20) | 1.01 (0.89-1.32) | 0.682 |
| Ku80 | 0.87 (0.78-0.99) | 0.96 (0.87-1.11) | **<0.001** |

Abbreviations: TERT=telomerase reverse transcriptase, T-klotho=total klotho, p16=cyclin dependent kinase inhibitor 2A, p21=cyclin dependent kinase inhibitor 1A, S-klotho=soluble klotho, TRF2=telomere repeat binding factor 2,Ku70=X-ray repair cross complementing 6, Ku80=X-ray repair cross complementing 5.

**Table E2. Correlation between the clinical variables and the relative telomere length, SIRT1 and Ku80 expression levels in patients with clinically stable bronchiectasis**

| **Variables** | **Spearman’s correlation coefficient, *P* value** | | |
| --- | --- | --- | --- |
|  | **Telomere length** | **SIRT1** | **Ku80** |
| **Age (yrs)** | **-0.308, <0.001** | **-0.139, 0.064** | **-0.197, 0.009** |
| **Sex** | 0.078, 0.303 | 0.019, 0.804 | 0.025, 0.740 |
| **Body-mass index (kg/m^2^)** | -0.061, 0.424 | 0.069, 0.362 | -0.041, 0.590 |
| **FEV_1_% predicted** | **0.146, 0.054** | 0.118, 0.119 | 0.015, 0.847 |
| **FEV_1_/FVC%** | **0.146, 0.053** | 0.050, 0.511 | 0.075, 0.318 |
| **C-reactive protein** | -0.131, 0.110 | -0.085, 0.298 | 0.032, 0.696 |
| **White blood cell (*10^9/L)** | 0.011, 0.893 | -0.060, 0.443 | 0.062, 0.425 |
| **SOD2 mRNA expression** | -0.081, 0.284 | -0.024, 0.754 | 0.087, 0.251 |
| **Catalase mRNA expression** | 0.016, 0.835 | **0.272, <0.001** | 0.042, 0.579 |

FEV_1_ = forced expiratory volume in one second; pred% = the percentage predicted; FVC= forced vital capacity; SOD2 = superoxide dismutase 2; SIRT1 = sirtuin 1

**Table E3. Baseline characteristics of the participants in Study 2**

| **Variables** | **Bronchiectasis**  **(n=36)** | **Disease controls**  **(n=32)** | **P value** |
| --- | --- | --- | --- |
| **Age (yrs)** | 44.1±14.8 | 57.3±10.3 | **<0.001** |
| **Sex, females n (%)** | 19 (52.8%) | 14 (43.8%) | 0.457 |
| **Body-mass index (Kg/m^2^)** | 20.1±2.9 | 23.2±4.0 | **<0.001** |
| **Never-smokers (%)** | 33 (91.7%) | 26 (75.0%) | 0.062 |
| **FEV_1_ %** | 59.7 (42.5-77.0) | 96.6 (87.4-111.4) | **<0.001** |
| **FEV_1_/FVC** | 72.6 (63.0-79.8) | 79.8 (77.2-87.1) | **<0.001** |
| **HRCT score** | 8.5 (6.0-11.8) | NA | NA |
| **Aetiology of bronchiectasis** |  |  | NA |
| Idiopathic | 26 (72.2%) | NA |  |
| Post-infectious (other than tuberculosis) | 4 (11.1%) | NA |  |
| Post-tuberculous | 6 (16.7%) | NA |  |
| **Idiopathic pulmonary fibrosis** | NA | 10 (31.3%) |  |
| **Peripheral lung tumor** | NA | 22 (68.7%) |  |
| **Lung lobes biopsied** |  |  | 0.113 |
| Left upper lobe | 7 (19.4%) | 8 (25.0%) |  |
| Left lower lobe | 12 (33.2%) | 4 (12.4%) |  |
| Right upper lobe | 1 (2.8%) | 6 (18.8%) |  |
| Right middle lobe | 7 (19.4%) | 6 (18.8%) |  |
| Right lower lobe | 9 (25.0%) | 8 (25.0%) |  |
| **Anatomical site of airways** |  |  | 0.378 |
| Large-to-medium airways | 30 (54.5%) | 22 (45.8%) |  |
| Small airways | 25 (45.5%) | 26 (54.2%) |  |

Abbreviations: FEV_1_ = forced expiratory volume in one second; pred% = the percentage predicted; FVC= forced vital capacity; HRCT = high-resolution computed tomography; NA = not applicable

Continuous variables were initially checked for normality, and expressed as mean ± standard or median (interquartile range) as appropriate. Categorical variables were summarized as count by (percentage).

**Table E4. The percentage of positively stained cells that corresponded to SIRT1, p16 and p21**

|  | **Bronchiectasis patients** | | **Disease controls** | | **P value** |
| --- | --- | --- | --- | --- | --- |
|  | **Median** | **Interquartile range** | **Median** | **Interquartile range** |  |
| **p16** | 40.0% | 32.0%-48.2% | 45.1% | 33.9%-54.5% | 0.113 |
| **SIRT1** | **25.1%** | **20.3%-30.6%** | **57.2%** | **38.1%-66.4%** | **<0.001** |
| **p21** | 28.9% | 22.8%-38.5% | 35.9% | 25.3%-42.7% | 0.113 |

SIRT1 = sirtuin1; Data in bold indicated the statistical analysis with significance.

**Table E5. Correlation between the clinical variables and the percentage of positively stained cells for senescence markers**

|  | **Age** | **Body-mass index** | **FEV_1_ pred%** | **FEV_1_/FVC%** |
| --- | --- | --- | --- | --- |
| **p16** | 0.121, 0.278 | -0.012, 0.921 | 0.201, 0.100 | 0.169, 0.168 |
| **Sirtuin1** | **0.231, 0.035** | 0.209, 0.073 | **0.294, 0.014** | 0.232, 0.055 |
| **p21** | 0.082, 0.457 | 0.175, 0.136 | **0.312, 0.011** | 0.135, 0.280 |

FEV_1_ = forced expiratory volume in one second; pred% = the percentage predicted; FVC= forced vital capacity

Shown are the correlation coefficient and the P values. Data in bold indicated the statistical analysis with significance.

**Figure legends**

**Figure E1. The expression levels of the relative telomere length, SIRT1 and Ku80 in the peripheral blood mononuclear cells between the 132 bronchiectasis patients and healthy controls**

Shown are the comparisons of the expression levels of the three differentially expressed senescence markers. This analysis was restricted to the single clinically stable samples collected at the initial visit only.

**Figure E2. Comparison of the expression levels of ageing markers within two clinically stable visits among the 32 bronchiectasis patients who had dual samples**

The horizontal axis demonstrates the mean of the first and the second measurement, whereas the vertical axis shows the difference in the expression levels between the two tests. ±1.96SD corresponds to the 95% confidence interval of the difference between the two tests.

**Figure E3. The expression levels and diagnostic performance of ageing markers in the peripheral blood mononuclear cells of bronchiectasis patients with different severity**

Panels A-D: Comparison of the expression levels of different ageing markers in the peripheral blood mononuclear cells of patients with mild-to-moderate bronchiectasis and those with severe bronchiectasis. A) SIRT1 expression levels; B) the relative telomere length, expressed as the T/S ratio; C) Ku70 expression levels; D) TERT expression levels;

The expression level of ageing markers was expressed as the fold-change by using the 2^-△△^ cycle threshold algorithm, with exception of the T/S ratio for the relative telomere length.

Panel E: The diagnostic performance of ageing markers and their combination to discriminate patients with mild-to-moderate bronchiectasis and those with severe bronchiectasis.

Combination denoted the receiver operation characteristics curve of the sum of the four ageing markers (SIRT1, relative telomere length, Ku70 and TERT).

AUC: area under curve; 95%CI: 95% confidence interval; Bx: Bronchiectasis; SIRT1: sirtuin 1; TERT: telomerase reverse transcriptase

**Figure E4. Expression of other senescence markers in bronchiectasis patients stratified by the disease severity**

The Bronchiectasis Severity Index was adopted for the severity classification into three categories: mild, moderate and severe

├┤ denoted three-group comparisons; ─ demonstrated two-group comparisons; * denoted P<0.05. Bonferroni algorithm was adopted for the *post hoc* between-group comparisons.
